# Gomisin N inhibits adipogenesis and prevents high-fat diet-induced obesity

**DOI:** 10.1038/srep40345

**Published:** 2017-01-09

**Authors:** Min-Kyung Jang, Ye-Rang Yun, Ji-Hyun Kim, Mi-Hee Park, Myeong Ho Jung

**Affiliations:** 1 Division of Longevity and Biofunctional Medicine, School of Korean Medicine, Pusan National University, Yangsan-si, Gyeongnam, South Korea; 2 Healthy Aging Korean Medical Research Center, School of Korean Medicine, Pusan National University, Yangsan-si, Gyeongnam, South Korea

## Abstract

Gomisin N (GN) is a physiological lignan derived from *Schisandra chinensis*. In the present study, we investigated the inhibitory effects of GN on differentiation of 3T3-L1 preadipocytes and the anti-obesity effects of GN in high-fat diet (HFD)-induced obese mice. Incubation with GN significantly inhibited the differentiation of 3T3-L1 preadipocytes in a dose-dependent manner. This inhibitory effect primarily occurred at an early adipogenic stage through impairment of mitotic clonal expansion (MCE) caused by cell cycle arrest at the G1/S phase transition. GN inhibited the extracellular signal-regulated kinase and phosphoinositide 3-kinase/protein kinase B signaling in the MCE process and activated AMP-activated protein kinase. Furthermore, GN downregulated CCAT/enhancer-binding protein β (C/EBPβ) and histone H3K9 demethylase JMJD2B during early stages of adipogenesis, and therefore repressed the expression of C/EBPβ-targeted cell cycle genes. In addition, GN also repressed the expression of histone H3K4 methyltransferase MLL4 and reduced PPARγ expression. Moreover, GN effectively lowered the final body weight, adipose tissue mass, and reduced the serum levels of glucose, total triglyceride, and cholesterol in the HFD-induced obese mice. GN also markedly reduced hepatic triglyceride level induced by HFD. Collectively, these findings suggest that GN has potential as a novel agent for the prevention and treatment of obesity.

Obesity is a major global health problem because it is associated with an increased risk of metabolic disorders, including type 2 diabetes mellitus, hypertension, atherosclerosis, cancer, and cardiovascular disease^1^. It occurs owing to an imbalance between energy intake and expenditure, resulting in subsequent excess accumulation of adipose tissue. The increase in adipose tissue is caused by an increase in adipocyte numbers as a result of increased proliferation and differentiation and by an increase in adipocyte sizes due to lipid accumulation^2^. Therefore, inhibition of adipocyte proliferation and differentiation could be an effective strategy for the treatment and prevention of obesity and its associated metabolic diseases.

The regulation of adipocyte differentiation (adipogenesis) by hormones and nutrition is generally well understood^3^,^4^,^5^. Adipocyte differentiation from fibroblastic preadipocytes is a multistep process consisting of three stages, growth arrest of confluent preadipocytes, mitotic clonal expansion (MCE), and terminal differentiation. Growth-arrested confluent preadipocytes re-enter the cell cycle in a differentiation medium cocktail, including insulin, dexamethasone, and 3-isobutyl-1-methylxanthine (IBMX). Then, they increase in number, exit the cell cycle, and undergo terminal differentiation into mature adipocytes. The increase in cell numbers during MCE is required for the activation of signaling pathways, such as the phosphoinositide 3-kinase/protein kinase B (PI3K/Akt)^6^ and mitogen- activated protein kinase (MAPK) pathways including extracellular signal-regulated kinase (ERK) and p38 MAPK^7^,^8^,^9^. Furthermore, several early adipogenic transcription factors, including CCAT/enhancer-binding protein β (C/EBPβ), C/EBPδ, and Kruppel-like factors 4 and 5, are transcriptionally activated during the MCE process^10^. These transcription factors stimulate master adipogenic transcription factors essential for terminal differentiation, such as peroxisome proliferator-activated receptor gamma (PPARγ) and C/EBPα. PPARγ and C/EBPα coordinately stimulate the expression of triglyceride synthesis genes, such as the genes for adipocyte fatty acid-binding protein 2 (aP2), fatty acid synthase (FAS), acetyl-coenzyme A carboxylase (ACC), lipin 1, and diacylglycerol acyltransferase. These proteins, associated with lipogenesis and tracylglycerol synthesis, induce the formation of lipid droplets in mature adipocytes. Other transcription factors, such as the glucocorticoid receptor, cyclic AMP response element-binding protein, and sterol regulatory element-binding protein-1c, also positively regulate the adipocyte differentiation^10^. On the contrary, the Wnt/β-catenin signaling pathway negatively regulates the adipocyte differentiation^10^.

The fruit of *Schisandra chinensis* has been used as a traditional herbal medicine in China, Korea, Japan, and Russia. Several studies have demonstrated the diverse pharmacological activities of *S. chinensis*, including antioxidant^11^, antitumor^12^, anti-obesity^13^, anti-inflammatory^14^, cardioprotective^15^, and hepatoprotective effects^16^. *S. chinensis* contains various bioactive constituents, including lignans, triterpenoids, polysaccharides, and sterols^17^. Gomisin N (GN) is a lignan from *S. chinensis* and was reported to exhibit hepatoprotective^18^, anticancer^19^, and anti-inflammatory effects^20^. Very recently, we have reported that *S. chinensis* and GN show protective effects against the endoplasmic reticulum (ER) stress-induced hepatic steatosis^21^,^22^.

Inhibition of adipocyte differentiation is an important strategy to prevent the initiation and progression of obesity, and therefore, an extensive search for natural agents that suppress this process has been undertaken. In this study, to examine whether GN has anti-obesity effects, we investigated the inhibitory effects of GN on differentiation of 3T3-L1 preadipocytes and explored the potential molecular mechanisms underlying these effects. Furthermore, we assessed the *in vivo* anti-obesity effects of GN in high-fat diet (HFD)-induced obese mice.

## Results

### Gomisin N inhibits differentiation of 3T3-L1 preadipocytes

**Figure 1:**
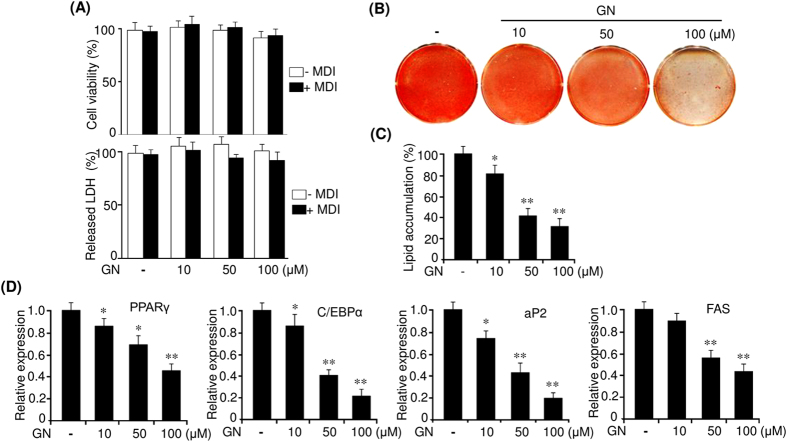
Gomisin N (GN) inhibits the differentiation of 3T3-L1 preadipocytes. (**A**) Post confluent 3T3-L1 preadipocytes (day 0) were incubated in the presence of various concentrations of GN (10 μM, 50 μM, 100 μM) with MDI or without MDI for 48 h. Cell viability were determined by the MTT assay. Cytotoxicity was assessed by measurement of LDH released in medium. (**B**) Post-confluent 3T3-L1 preadipocytes were differentiated in MDI medium containing various concentrations of GN, and adipocyte differentiation was examined on day 8 by ORO staining. (**C**) Intracellular triglyceride accumulation was measured on day 8 of differentiation. (**D**) Expression of PPARγ, C/EBPα, aP2, and FAS was measured on day 8 by qPCR. The qPCR data are presented as the mean ± SEM of three replicate experiments. *P < 0.05, **P < 0.01 vs. no GN treatment.

We first examine the effects of GN on the viability and cytotoxicity of 3T3-L1 cells. Post confluent 3T3-L1 preadipocytes were incubated in the presence of various concentrations of GN with the differentiation medium (MDI) or without MDI for 48 h. The MTT assay showed that GN did not affect the viability of both 3T3-L1 preadipocytes and differentiating 3T3-L1 adipocytes up to a concentration of 100 μM (Fig. 1A). Consistently, we also observed that GN exerted no cytotoxic effects on both 3T3-L1 adipocytes cells as assessed by the lactate dehydrogenase (LDH) assay (Fig. 1A). Then, we investigated the inhibitory effects of GN on adipocyte differentiation. 3T3-L1 preadipocytes were differentiated in the MDI medium with the indicated concentrations of GN for eight days, and accumulation of intracellular lipid droplets was assessed by Oil Red O (ORO) staining. As shown in Fig. 1B, the ORO staining revealed that GN dose-dependently inhibited the lipid accumulation. In particular, 100 μM GN greatly suppressed the formation of lipid droplets in the adipocytes, as compared to that in MDI only-treated cells, without affecting cell viability. The measurement of the intracellular triglyceride content indicated that GN also reduced intracellular triglycerides in a dose-dependent manner (Fig. 1C). To confirm the inhibition of adipocyte differentiation, we measured the expression of adipogenic genes, such as PPARγ and C/EBPα, as well as their target genes (aP2 and FAS), by quantitative PCR (qPCR). As shown in Fig. 1D, GN dose-dependently decreased the mRNA levels of PPARγ, C/EBPα, aP2, and FAS. These results demonstrate that GN inhibits the differentiation of 3T3-L1 preadipocytes.

### Gomisin N mostly inhibits early stages of adipocyte differentiation through attenuation of mitotic clonal expansion

**Figure 2:**
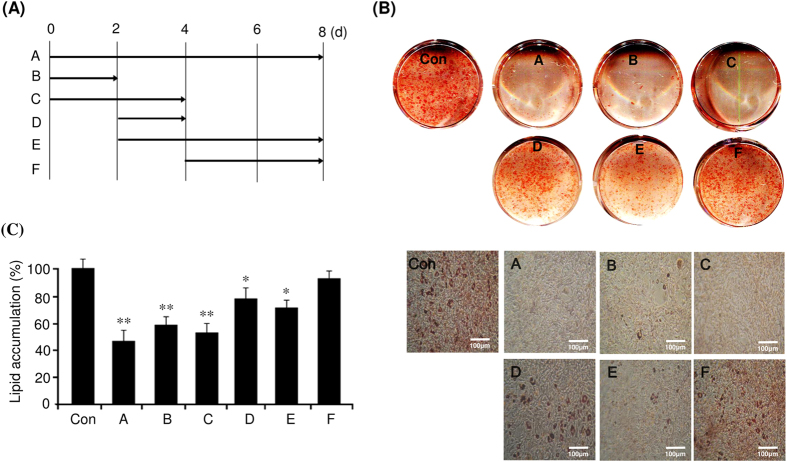
Gomisin N (GN) mostly inhibits the early stage of adipocyte differentiation. (**A**) Post-confluent 3T3-L1 preadipocytes (day 0) were differentiated in MDI and treated with 100 μM GN on different days ((**A**), 0–8; (**B**), 0–2; C, 0–4; D, 2–4; E, 2–8; F, 4–8). (**B**) After eight days of differentiation, adipocyte differentiation was examined by ORO staining (scale bar, 100 μm). (**C**) Intracellular triglyceride accumulation was measured on day 8 of differentiation. The data are presented as the mean ± SEM of three replicate experiments. *P < 0.05, **P < 0.01 vs. no GN treatment. Con means no GN treatment.

**Figure 3:**
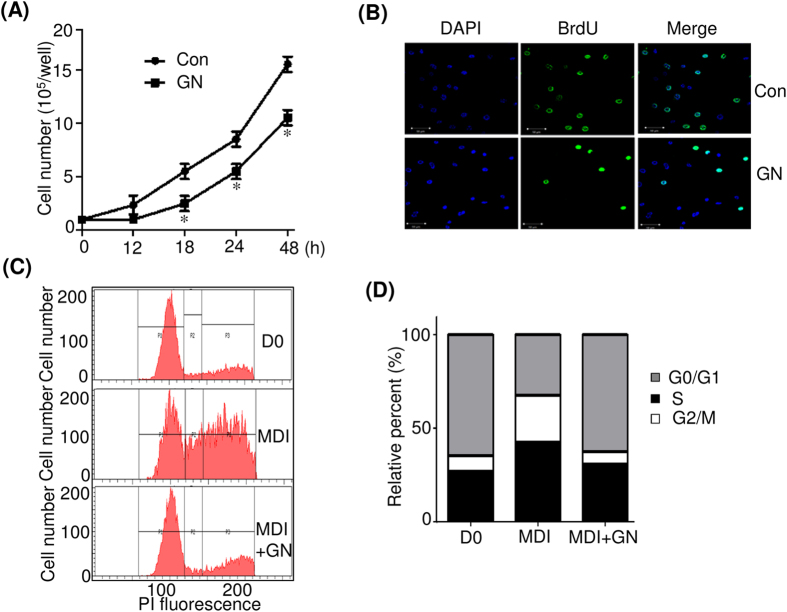
Gomisin N (GN) inhibits the cell cycle progression during mitotic clonal expansion. (**A**) Post-confluent 3T3-L1 preadipocytes (day 0) were differentiated in MDI with or without 100 μM GN, and cell numbers were determined at the indicated times. *P < 0.05 vs. no GN treatment. (**B**) Post-confluent 3T3-L1 preadipocytes were differentiated in MDI with or without 100 μM of GN in the presence of BrdU for 24 h. Cells that incorporated BrdU were observed under a fluorescence microscope (scale bar, 50 μM). (**C**) Post-confluent 3T3-L1 preadipocytes were differentiated in MDI medium with or without 100 μM GN for 24 h, then harvested, fixed, and stained with propidium iodide (PI). The stained DNA was analyzed by flow cytometer. (**D**) The cell population at each stage of the cell cycle was determined using the BD Pro software. The results were of three independent experiments (n = 3). Con means no GN treatment.

Differentiation of 3T3-L1 preadipocytes is divided into three stages, an early stage (days 0–2), a postmitotic intermediate stage (days 3–4), and a terminal stage (after day 4)^4^. Therefore, we investigated the critical stage of adipocyte differentiation, specifically affected by GN treatment. 3T3-L1 cells were treated with 100 μM GN at various time points after induction of differentiation with MDI, as illustrated in Fig. 2A, and the levels of adipogenesis were determined by ORO staining. As shown in Fig. 2B, the treatment with GN at early adipogenic stages (days 0–2 and 0–4) significantly inhibited adipogenesis, and this effect was almost similar to that of a continuous treatment (days 0–8) (Fig. 2B). The treatment with GN after day 2 (days 2–4 and 2–8) showed moderate inhibition of adipogenesis, whereas the treatment after day 4 showed only a weak inhibitory effect (Fig. 2B). These results were also confirmed by the measurement of the intracellular triglyceride content (Fig. 2C), which indicated that the GN inhibition of adipocyte differentiation mostly occurred at an early adipogenic stage. In the early adipogenic stage, the number of adipocytes greatly increases due to MCE^4^. Therefore, to determine whether GN affects MCE, we examined the effects of GN on proliferation of 3T3-L1 cells after induction of differentiation with MDI. As shown in Fig. 3A, the treatment with GN significantly decreased the cell numbers of MDI-treated 3T3-L1 adipocytes during day 2, as compared to the cell numbers of control adipocyte cells not treated with GN. Consistent with these results, treatment of 3T3-L1 preadipocytes with GN (100 μM) resulted in reduced levels of bromodeoxyuridine (BrdU) incorporation into cells compared to those of non-treated control adipocyte cells (Fig. 3B), indicating that GN inhibits proliferation of 3T3-L1 cells in the MCE process. Next, to assess whether GN regulates the cell cycle progression during the MCE process, we examined the cell cycle distribution in GN-treated 3T3-L1 cells by flow cytometry analysis after induction with MDI. The flow cytometry and BD Pro software analyses revealed that the induction of differentiation of 3T3-L1 preadipocytes with MDI decreased the cell population in the G0/G1 phase but increased that in the S phase, indicating that the MDI treatment induced the normal cell cycle progression from the G0/G1 phase to the S and G2/M phases (Fig. 3C,D). However, the treatment with GN significantly increased the G0/G1 cell population and concomitantly decreased the cell numbers in the S and G2/M phases (Fig. 3C,D), demonstrating that GN caused cell cycle arrest at the G0/G1 phase during the MCE process and delayed cell cycle progression into the S phase.

### Gomisin N inhibits MCE through downregulation of cyclins A and D and their partners CDK2 and CDK6

**Figure 4:**
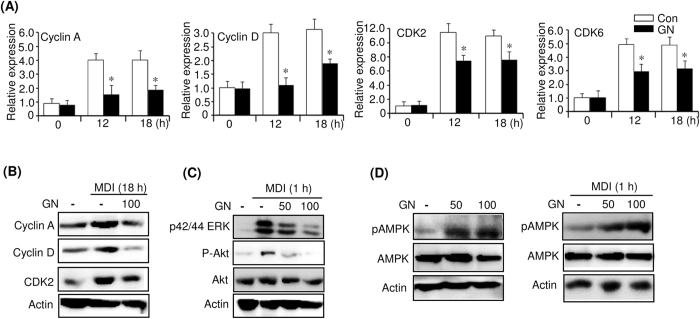
Gomisin N (GN) downregulates cyclin A, cyclin D, CDK2, CDK6 and inhibits ERK, PI3/Akt signaling, and activates AMPK. Post-confluent 3T3-L1 preadipocytes (day 0) were differentiated in MDI medium with or without 100 μM GN.(**A**) Expression of the cell cycle regulators was determined at the indicated times by qPCR. The qPCR data are presented as the mean ± SEM of three independent experiments. *P < 0.05 vs. no GN treatment. (**B**) After 18 h of differentiation with MDI, the protein levels of cyclin A, cyclinD, and CDK2 were measured by western blot. (**C**) Post-confluent 3T3-L1 preadipocytes (day 0) were differentiated in MDI medium with or without 50 μM or 100 μM GN for 1 h, and the phosphorylation levels of ERK and Akt were analyzed by western blot. (**D**) 3T3-L1 preadipocytes were treated with 50 μM or 100 μM GN for 1 h (left). Post-confluent 3T3-L1 preadipocytes (day 0) were differentiated in MDI with or without 50 μM or 100 μM GN for 1 h (right), and the phosphorylation level of AMPK was analyzed by western blot. The western blots were performed three times, and a representative image of three independent experiments was shown. Con means no GN treatment.

Cell cycle progression is regulated by cyclin-dependent kinases (CDKs) and their associated corresponding regulatory cyclins^23^. To examine whether these cell cycle regulators are affected by GN, we measured their expression in GN (100 μM)-treated 3T3-L1 cells by qPCR. As shown in Fig. 4A, induction of 3T3-L1 preadipocytes with MDI increased the mRNA levels of cyclins A and D, as well as those of CDK2 and CDK6, at 12 and 18 h. However, the treatment with GN significantly reduced these increased mRNA levels. Western blotting also showed that the GN treatment reduced the MDI-induced protein levels of cyclin A, cyclin D, and CDK2 at 18 h (Fig. 4B). These results suggest that GN inhibited the MDI-mediated MCE through downregulation of cyclin A, cyclin D, CDK2, and CDK6, which leads to a delay of entry into the S and G2 phases of the cell cycle. These results supported the data obtained by the flow cytometry analysis.

### Gomisin N inhibits activation of ERK and PI3K/Akt and activates AMPK

Intracellular mitogen-activated protein kinases (MAPKs), including ERK, c-Jun N-terminal kinase, and p38 MAPK, and the PI3K/Akt signaling pathway play major roles in the regulation of cell proliferation and differentiation^6^,^7^. Furthermore, it has been shown that the activation of ERK is essential for the induction of MCE and adipogenesis^8^. To further elucidate the mechanism underlying the inhibition of adipogenesis by GN, we investigated the signaling pathways involved. To this end, we measured by western blot the phosphorylation of ERK and Akt in GN (100 μM)-treated 3T3-L1 cells after induction of preadipocytes with MDI. Induction of 3T3-L1 preadipocytes for 1 h considerably increased the phosphorylation of ERK and Akt (Fig. 4C). However, the treatment with GN decreased the phosphorylation in a dose-dependent manner (Fig. 4C), indicating that GN inhibits the cell proliferation in the MCE process through suppression of ERK and Akt signaling. Furthermore, it has been reported that AMP-activated protein kinase (AMPK) inhibits the differentiation of 3T3-L1 preadipocytes through impairment of MCE and downregulation of several adipocyte-specific transcription factors^24^,^25^,^26^. Therefore, to examine whether GN activates AMPK, we assessed the AMPK phosphorylation in GN-treated 3T3-L1 preadipocytes. As shown in Fig. 4D, GN treatment increased the phosphorylation of AMPK in 3T3-L1 preadipocytes (Fig. 4D, left). We also examined the AMPK phosphorylation in 3T3-L1 cells treated with GN (100 μM) after induction of differentiation with MDI for 1 h. GN treatment also increased the phosphorylation of AMPK in MDI-treated 3T3-L1 cells in a dose-dependent manner (Fig. 4D, right). Taken together, these results suggest that AMPK activation, as well as the inhibition of ERK and PI3K/Akt signaling, may be involved in the GN-mediated inhibition of MCE.

### Gomisin N represses expression of early adipogenic factor C/EBPβ and C/EBPβ-targeted cell cycle genes during MCE

**Figure 5:**
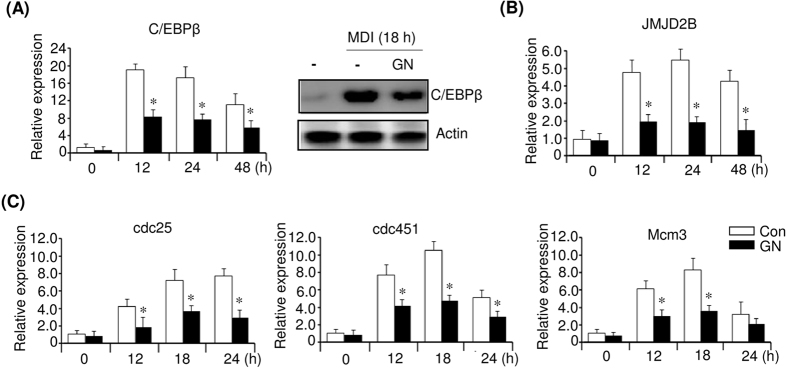
Gomisin N (GN) represses the expression of C/EBPβ and C/EBPβ-targeted cell cycle genes. Post-confluent 3T3-L1 preadipocytes (day 0) were differentiated in MDI medium with or without 100 μM GN. (**A**) mRNA levels of C/EBPβ were determined at the indicated times by qPCR, and its protein level was determined at 18 h by western blot. (**B**) Expression of JMJD2B was determined at the indicated times by qPCR. (**C**) Expression of C/EBPβ-targeted cell cycle genes was determined at the indicated times by qPCR. The qPCR data are presented as the mean ± SEM of three replicate experiments. *P < 0.05 vs. no GN treatment. Con means no GN treatment.

The early adipogenic factor C/EBPβ, which is expressed early during adipocyte differentiation, is required for both MCE and the stimulation of the PPARγ and C/EBPα expression^27^,^28^,^29^. To further assess the regulators involved in the GN inhibition of MCE, we investigated the expression of C/EBPβ in GN (100 μM)-treated 3T3-L1 cells at the indicated times after MDI induction. As shown in Fig. 5, the level of C/EBPβ mRNA was increased by the MDI treatment during MCE; however, GN treatment effectively decreased the C/EBPβ mRNA level (Fig. 5A, left). Western blotting also showed that GN reduced the MDI-induced C/EBPβ protein level at 18 h (Fig. 5A, right). A recent study has shown that C/EBPβ stimulated the expression of histone H3K9 demethylase JMJD2B and upregulated cell cycle genes, including *Cdc45l, Mcm3*, and *Cdc25c*, through decreasing H3K9me3 in their regulatory regions^30^. Therefore, we measured the expression of JMJD2B and the cell cycle genes in GN (100 μM)-treated 3T3-L1 cells during the MCE process. Concomitant with the reduced C/EBPβ expression, the JMJD2B mRNA level was also reduced by GN treatment (Fig. 5B). Consequently, the reduction of C/EBPβ and JMJD2B mRNA expression finally led to the repression of mRNA transcription of the cell cycle genes *Cdc25c, Cdc45l*, and *Mcm3* during MCE (Fig. 5C). These results suggested that the GN inhibition of MCE might be epigenetically regulated through downregulation of C/EBPβ and JMJD2B.

### Gomisin N downregulates H3K4 methyltransferase MLL4, which may be involved in gomisin N-mediated inhibition of PPARγ expression

**Figure 6:**
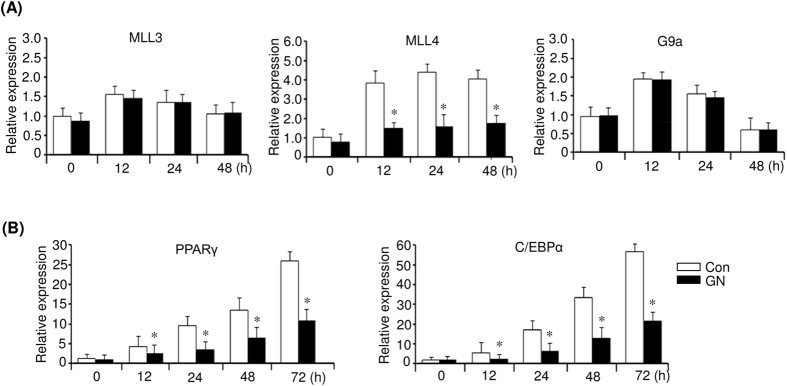
Gomisin N (GN) represses the expression of histone H3K4 methyltransferase MLL4. Post-confluent 3T3-L1 preadipocytes (day 0) were differentiated in MDI medium with or without 100 μM GN. (**A**) Expression of MLL3, MLL4, and G9a was determined at the indicated times by qPCR. (**B**) Expression of PPARγ and C/EBPα was determined at the indicated times by qPCR. The qPCR data are presented as the mean ± SEM of three replicate experiments. *P < 0.05 vs. no GN treatment. Con means no GN treatment.

Several histone methyltransferases and demethylases have been reported to regulate adipogenesis^31^,^32^,^33^,^34^,^35^. Among those, H3K4 me/me2 methyltransferases MLL3 and MLL4 stimulate PPARγ expression^31^,^32^, whereas H3K9 me/me2 methyltransferase G9a represses PPARγ expression during adipogenesis^33^. Therefore, we examined whether GN affects the expression of these histone methyltransferases during adipogenesis. To this end, we investigated the expression of MLL3, MLL4, and G9a in GN-treated 3T3-L1 cells at the indicated times after MDI induction. As shown in Fig. 6A, GN effectively repressed the MLL4 mRNA expression during early adipogenesis but did not repress that of MLL3 and G9a. Consistent with the reduced MLL4 expression, the levels of PPARγ and C/EBPα mRNA also decreased in GN-treated 3T3-L1 cells (Fig. 6B).

### Gomisin N prevents HFD-induced obesity and ameliorates hepatic steatosis

**Figure 7:**
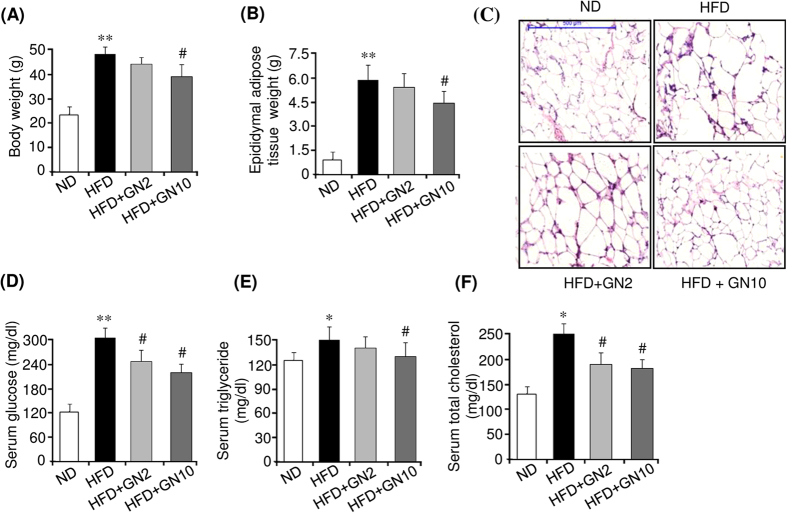
Gomisin N (GN) prevents HFD-induced obesity and ameliorates serum biochemical parameters. C57BL/6 mice were fed a normal diet (ND) or high-fat diet (HFD) for six weeks, and a low or high dose of GN was administered to the HFD-fed mice for an additional eight weeks. (**A**) Final body weight. (**B**) Final epididymal adipose tissue weight. (**C**) H&E staining (scale bar, 500 μm) (**D**) Serum glucose level. (**E**) Serum triglyceride level. (**F**) Serum total cholesterol level. The data are presented as the mean ± SEM for six mice. *P < 0.05, **P < 0.01 vs. ND fed mice. ^*#*^*P* < 0.05 vs. HFD fed mice alone.

**Figure 8:**
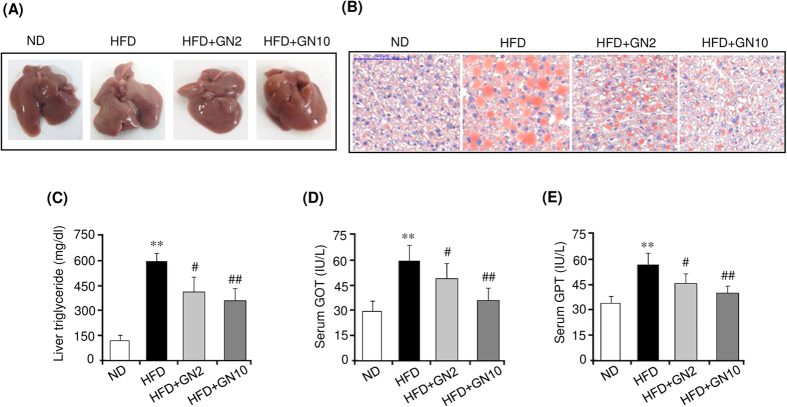
Gomisin N (GN) ameliorates hepatic steatosis in HFD-induced obese mice. (**A**) Liver morphology. (**B**) ORO staining (scale bar, 100 μm). (**C**) Measurement of liver triglycerides. (**C**) Measurement of GOT and GPT levels. The data are presented as the mean ± SEM for six mice. **P < 0.01 vs. ND fed mice. ^*#*^*P* < 0.05, ^#*#*^*P* < 0.01 vs. HFD fed mice alone.

We further investigated the anti-obesity effects of GN in HFD-induced obese mice. To this end, 6-week-old mice were fed a high fat diet (HFD) or normal diet (ND) for six weeks, and the HFD-fed mice were further administered a low (2 mg/kg of body weight) or high dose (10 mg/kg of body weight) of GN for eight weeks. As shown in Fig. 7, HFD feeding for 14 weeks significantly increased the body weight (Fig. 7A) and epididymal adipose tissue weight (Fig. 7B) compared to those in the ND mice. However, the high dose of GN effectively lowered both the body weight and epididymal adipose tissue weight. Hematoxylin and eosin (H&E) staining also showed that HFD feeding resulted in increased average adipocyte size in epididymal adipose tissue, compared to average adipocyte size in the ND mice, whereas the high dose of GN administration efficiently reduced adipocyte enlargement (Fig. 7C). These results demonstrated that GN could potentially prevent the HFD-induced body weight gain and adiposity. Furthermore, we examined the serum biochemical profiles of HFD-induced obese mice to confirm the anti-obesity effects of GN. Compared to the ND mice, the mice fed an HFD had significantly higher serum levels of glucose (Fig. 7D), total triglycerides (Fig. 7E), and total cholesterol (Fig. 7F). However, the high dose of GN efficiently reduced the serum levels of glucose, triglycerides, and total cholesterol, indicating that GN administration improved glucose and lipid homeostasis in the HFD-induced obese mice. In addition, we investigated the expression of adipogenic factors in the epididymal adipose tissue of HFD-induced obese mice. GN effectively reduced the mRNA levels of PPARγ, C/EBPα, aP2, and FAS, which were induced by HFD feeding (Supplemental Fig. S1). Furthermore, we investigated the anti-hepatic steatosis effects of GN on the liver of HFD-induced obese mice, in which the HFD induced hepatic steatosis. As shown in Fig. 8A, the HFD resulted in a white-colored fatty liver in the mice. However, administration of the high dose of GN to the HFD-fed mice converted the white-colored fatty liver into a relatively healthy liver. ORO staining (Fig. 8B) and the measurement of hepatic triglycerides (Fig. 8C) also showed that HFD feeding elevated triglycerides in the liver of the HFD-fed mice, but the GN treatment blocked the elevation of hepatic triglycerides. In addition, we examined whether GN improves hepatic injuries induced by the HFD. As shown in Fig. 8D,E, the serum levels of glutamate oxaloacetate transaminase (GOT) (Fig. 8D) and glutamate pyruvate transaminase (GPT) (Fig. 8E) were significantly increased by HFD feeding, whereas GN significantly decreased the levels. All these results suggest that GN ameliorated hepatic steatosis and hepatic injuries in the HFD-induced obese mice.

## Discussion

The inhibition of adipocyte differentiation has been recognized as a potential target for many plant extracts and bioactive compounds tested for the prevention and treatment of obesity. It has been reported that several phytochemicals, including epigallocatechin-3-gallate^36^ and resveratrol^37^, inhibited the differentiation of preadipocytes to adipocytes and thereby exhibited anti-obesity effects^38^. GN is a phytochemical lignan isolated from *S. chinensis*^17^. Previously, we have reported that GN showed a protective effect against ER stress-induced hepatosteatosis^22^. In the present study, we demonstrated that GN inhibited the differentiation of 3T3-L1 preadipocytes, primarily through the impairment of the MCE process, during the early stage of adipocyte differentiation. Furthermore, we showed that the administration of GN to HFD-fed mice resulted in a less obese phenotype, reduced adiposity and hepatic triglyceride.

Adipocyte differentiation occurs via three stages, the growth arrest of confluent preadipocytes, MCE, and terminal differentiation^3^,^4^,^5^. After induction of differentiation with MDI, the growth-arrested preadipocytes synchronously re-enter the cell cycle for two additional rounds of division. The cells progress from the G0/G1 to S and G2/M phases at 24 h, and cell proliferation increases, which is known as the MCE process during the early stage of adipocyte differentiation. Our current data revealed that GN treatment suppressed the early stage of adipogenesis through inhibition of MCE, which was evidenced by the impaired cell proliferation and delayed cell-cycle entry into the S and G2/M phases. Here, we propose several mechanisms by which GN inhibits MCE during the differentiation of 3T3-L1 preadipocytes. CDKs and their associated regulatory cyclins play a key role in the regulation of cell cycle processes^23^. The G1/S transition is regulated by complexes formed between cyclin D (G1 phase regulator) and CDK4 or CDK6. In addition, cyclin A (S phase regulator) and its partner CDK2 are responsible for the S phase progression. Therefore, cyclins A and D are essential for the progression of the cell cycle from the G0/G1 phase to the S and G2/M phases. Our results revealed that MDI induction resulted in an increase in the expression of cyclin D, cyclin A, CDK2, and CDK6, whereas GN treatment considerably reduced their expression, which was associated with delayed G0/G1 to S phase transition and subsequent transition to the G2/M phase. These results were consistent with those obtained by flow cytometry analysis, which showed that the induction of differentiation of 3T3-L1 cells with MDI increased, whereas GN treatment significantly decreased, the cell numbers in the S and G2/M phases. Thus, the downregulation of cyclin A, cyclin D, CDK2, and CDK6 may contribute to the GN inhibition of MCE through the delayed entry of G0/G1 cells into the S phase. It has been reported that intracellular MAPK and PI3K/Akt signaling pathways play major roles in the regulation of cell proliferation and differentiation^6^,^7^,^8^. Our data also showed that the induction of differentiation with MDI induced phosphorylation of ERK and Akt. However, GN treatment inhibited the activation of ERK and Akt in a dose-dependent manner, indicating that GN inhibits the cell proliferation during MCE through the suppression of ERK and Akt signaling.

It has been reported that several AMPK-activating compounds inhibit adipogenesis via the impairment of MCE and suppression of adipogenic transcriptional pathways^24^,^25^,^26^. Therefore, to further elucidate the mechanism involved in the GN-induced inhibition of MCE, we investigated AMPK activation in both 3T3-L1 preadipocytes and 3T3-L1 cells treated with MDI. GN treatment increased the phosphorylation of AMPK in 3T3-L1 preadipocytes. Furthermore, the treatment with GN also increased the phosphorylation of AMPK in 3T3-L1 cells treated with MDI for 1 h, suggesting that GN stimulates the AMPK activation in 3T3-L1 cells. A recent study has suggested the role for the AMPK/mammalian target of rapamycin complex 1 (mTORC1) axis in the AMPK-mediated inhibition of cell cycle progression^39^. mTORC1 promotes the cell growth and cell cycle progression, which drive the cell proliferation. Under the condition that AMPK is activated, AMPK phosphorylates tuberous sclerosis complex 2 (TSC2) and increases the activity of the TSC1–TSC2 complex to inhibit mTOR. Furthermore, AMPK also phosphorylates the mTORC1 component raptor, leading to the 14-3-3 protein binding and allosteric inhibition of mTORC1. Therefore, the phosphorylation of TSC2 and mTORC1 causes the inhibition of mTORC1 activity, which results in the suppression of cell growth and cell cycle progression. Accordingly, GN-activated AMPK may also be involved in the inhibition of MCE through the suppression of mTORC1 activity. The effect of GN on the AMPK/mTORC1 axis in 3T3-L1 cells should be further investigated.

C/EBPβ plays an essential role in the differentiation of 3T3-L1 preadipocytes^27^,^28^,^29^. During the 3T3-L1 adipocyte differentiation, C/EBPβ is induced early to promote the expression of PPARγ and C/EBPα for terminal adipocyte differentiation. Very recently, a ChIP-on-chip combined gene expression microarray has allowed identification of new C/EBPβ-targeted cell cycle genes in the MCE process^28^. It has been shown that C/EBPβ stimulates the expression of histone H3K9 demethylase JMJD2B, which functions as a co-factor of C/EBPβ to demethylate H3K9me3 in the regulatory regions of C/EBPβ-regulated cell cycle genes, including *Cdc45l, Mcm3, Gins1*, and *Cdc25c*, thereby promoting their expression and MCE. Our results showed that GN treatment efficiently decreased the expression of both C/EBPβ and JMJD2B in the MCE process and, therefore, repressed the expression of *Cdc25c, Cdc45l*, and *Mcm3*. These results suggested that the GN-mediated downregulation of both C/EBPβ and JMJD2B might also contribute to the inhibition of MCE through the repression of cell cycle genes in the MCE process. A further study of the GN-mediated histone H3K9 enrichment in the regulatory regions of C/EBPβ-targeted cell cycle genes should be performed. In addition, our data revealed that GN downregulated the CDK2 expression in the MCE process. Since CDK2 has been reported to phosphorylate and activate C/EBPβ^40^, GN could inhibit MCE during early adipogenesis through blocking the CDK2-mediated activation of C/EBPβ.

It has been demonstrated that MLL4 is essential for the PPARγ expression and adipogenesis^31^,^32^. During adipogenesis, MLL4 is mainly bound to adipogenic active enhancers bound by PPARγ, C/EBPα, and C/EBPβ^32^. In the early stage of adipogenesis, C/EBPβ recruits MLL4 to perform H3K4 mono- and dimethylation at the enhancers on the PPARγ and C/EBPα genes and thereby stimulates their expression. Therefore, knockdown of MLL4 prevents the activation of adipogenic enhancers on the PPARγ and C/EBPα genes, which leads to severe defects in adipogenesis. In the present study, we observed that GN substantially repressed the MLL4 expression, as well as the expression of PPARγ and C/EBPα, suggesting that the GN-mediated repression of MLL4 results in downregulation of PPARγ and C/EBPα. The H3K4 mono-/dimethylation at the enhancers on the PPARγ and C/EBPα genes should also be characterized in GN-treated 3T3-L1 cells.

In addition, we investigated the *in vivo* anti-obesity effects of GN in HFD-induced obese mice. Compared to the ND mice, the HFD-fed mice showed a higher final body weight and epididymal adipose tissue weight, as well as higher serum levels of glucose and triglycerides. However, administration of a high dose of GN significantly lowered the final body weight, epididymal adipose tissue weight, and serum levels of glucose and triglycerides, with no significant change in the food intake (Supplemental Fig. S2), demonstrating that GN can prevent the HFD-induced obesity and ameliorate the serum metabolic parameters. Furthermore, GN administration also reduced the HFD-induced hepatic triglyceride and the serum levels of GOT and GPT, indicating that GN improved hepatic steatosis and hepatic injuries in the HFD-induced obese mice. The *de novo* adipogenesis of small adipocyte reduces insulin resistance in obesity and enhances insulin-dependent glucose uptake, which exerts beneficial effects on metabolic parameters^41^. However, although GN inhibited *in vitro* adipogenesis, the results of our present study revealed that GN ameliorated serum metabolic parameters including blood glucose, triglycerides, and total cholesterol. AMPK regulates glucose and lipid metabolism in the liver and skeletal muscle^42^. In the liver, AMPK stimulates fatty-acid oxidation by inhibiting ACC through phosphorylation and activation of the expression of fatty acid oxidation genes such as carnitine palmitoyltransferase1. AMPK also inhibits the synthesis of triglyceride and cholesterol by suppressing the expression of lipogenesis genes including sterol regulatory element binding protein1c, FAS, and HMGCoA reductase. Furthermore, AMPK inhibits gluconeogenesis by suppressing gluconeogenesis genes such as phosphoenolpyruvate carboxykinase and glucose-6-phosphatase. In skeletal muscle, AMPK stimulates glucose uptake through increased translocation and expression of glucose transporter-4, and increases fatty acid oxidation and mitochondrial biogenesis. These metabolic effects of AMPK exert antidiabetic and anti-hepatosteatotic actions. In the present study, we demonstrated that GN activated AMPK in 3T3-L1 adipocyte as shown in Fig. 4D. Furthermore, we also observed that GN activated AMPK in HepG2 cells, and inhibited the expression of lipogenesis genes and stimulated the expression of fatty acid oxidation and mitochondrial genes in HepG2 cells. In addition, GN inhibited the liver X-receptor (LXR) or palmitate (PA)-induced lipogenesis in HepG2 cells, and prevented intracellular triglyceride accumulation induced by LXR and PA (unpublished data). These effects of GN may have contributed to the anti-hepatosteatotic effects observed in the GN-administered HFD mice. Therefore, GN-mediated stimulation of AMPK activation may play a role in the beneficial effects of GN on metabolic parameters in HFD obese mice.

In summary, GN effectively inhibited the differentiation of 3T3-L1 preadipocytes through the impairment of the MCE process during the early stage of adipogenesis via suppression of cell proliferation and cell cycle progression. The downregulation of cyclins A and D, CDK2, and CDK6, the inhibition of ERK and PI3K/Akt signaling, and the AMPK activation may be involved in the GN inhibition of MCE. Furthermore, GN downregulated both C/EBPβ and JMJD2B in MCE and repressed the expression of C/EBPβ-targeted cell cycle genes, which may be also involved in the GN inhibition of MCE. In addition, GN repressed the MLL4 expression, which may contribute to the suppression of PPARγ and C/EBPα expression. Finally, administration of GN to HFD-fed mice reduced the final body weight gain, fat pad weight, adipocyte sizes, serum levels of glucose, triglyceride and hepatic triglyceride in the HFD-induced obese mice. Based on these results, we conclude that GN has a great potential as a novel agent for the prevention and treatment of obesity.

## Materials and Methods

### Reagents

GN was purchased from ChemFaces (Wuhan, China). Dulbecco’s modified Eagle’s medium (DMEM), penicillin–streptomycin, and fetal bovine serum (FBS) were obtained from Gibco BRL (Grand Island, NY, USA). Insulin, IBMX, dexamethasone, and rosiglitazone were purchased from Sigma Aldrich (St. Louis, MO, USA). Antibodies against p-42/44 ERK, phospho-Akt (S473), Akt, phospho-AMPKα (T172), and AMPKα were obtained from Cell Signaling Technology (Danvers, MA, USA). Antibodies against cyclin A, cyclin D, CDK2, C/EBPβ, and β-actin were purchased from Santa Cruz Biotechnology (Santa Cruz, CA, USA).

### Cell culture and differentiation of 3T3-L1 preadipocytes

3T3-L1 preadipocyte cells, purchased from the American Type Culture Collection (Manassas, VA, USA), were maintained in DMEM containing 10% FCS. Post-confluent 3T3-L1 preadipocytes (defined as day 0) were incubated in differentiation medium (MDI) containing DMEM, 10% FBS, 0.5 mM IBMX, 1 μM dexamethasone, 5 μg/mL insulin, and 2 mM rosiglitazone. After two days, the medium was replaced with DMEM containing 10% FBS and 5 μg/mL insulin, and the medium was changed every two days. The cells were fully differentiated into mature adipocytes on days 6–8.

### Cell viability and cytotoxicity assays

Briefly, post confluent 3T3-L1 preadipocytes (D0) were incubated in the presence of various concentrations of GN (10 μM, 50 μM, 100 μM) with MDI or without MDI for 48 h. The viability was determined using a 3-(4,5-dimethylthiazol-2-yl)-2,5-diphenyltetrazolium bromide (MTT) assay according to the manufacturer’s instructions (Promega, Madison, WI, USA). Cytotoxicity was measured using lactate dehydrogenase (LDH) cytotoxicity assay according to the manufacturer’s instructions (Promega).

### Oil Red O staining

3T3-L1 cells were washed twice with phosphate-buffered saline (PBS) and fixed with 10% formalin for 30 min. After fixation, the cells were washed with 60% isopropanol for 5 min and then stained with an Oil Red O (ORO) working solution (1.5 mg/mL ORO/60% isopropanol) for 15 min at room temperature. The cells were washed with distilled water and photographed under a light microscope.

### Measurement of triglyceride levels

3T3-L1 cells were harvested and washed twice with PBS. The cell suspensions were mixed with 750 μL of chloroform/methanol/H2O (8:4:3, v/v/v) to extract triglycerides. Adipose and liver tissues were homogenized in chloroform-methanol solution (2:1, v/v) and choloroform-methanol-H_2_O solution (8:4:3, v/v), respectively. The cell suspensions or the homogenates were incubated at room temperature for 1 h and centrifuged at 800 × g for 10 min. The obtained bottom layer (organic phase) was dried overnight, and then dissolved in ethanol, followed by determination of triglyceride concentrations using an enzyme reaction kit (Asan pharmaceutical, Seoul, South Korea) and normalized to the protein concentration.

### Quantitative polymerase chain reaction (qPCR)

Total RNA was extracted using TRIzol^®^ (Invitrogen, Paisley, Scotland) according to the manufacturer’s instructions, and DNA was digested using DNase I (Sigma). Total RNA was quantified by absorption measurements at 260 nm using a spectrophotometer. cDNA was generated from 1 μg of total RNA using the GoScript™ reverse transcription system (Promega) according to the manufacturer’s protocol. Polymerase chain reaction (PCR) amplification was performed using a SYBR Green premixed Taq reaction mixture with gene-specific primers. The primers used in this study are listed in Supplementary Table S1.

### Western blotting

3T3-L1 cells were harvested and lysed in ice-cold lysis buffer containing a protease inhibitor cocktail and 1 mM phenylmethanesulfonyl fluoride for 30 min, followed by centrifugation at 10,000 × g for 30 min at 4 °C. Proteins (50 μg) were subjected to sodium dodecyl sulfate polyacrylamide gel electrophoresis and then transferred to polyvinylidene difluoride membranes (Amersham Pharmacia Biotech, Amersham, UK). The membranes were incubated with primary antibodies, followed by incubation with anti-rabbit or anti-mouse secondary antibodies (Santa Cruz Biotechnology) and protein bands were visualized using an enhanced chemiluminescence system (ECL Advance, GE Healthcare, Hatfield, UK).

### Cell cycle analysis

3T3-L1 cells were harvested and fixed with 70% ice-cold ethanol at 4 °C for 24 h. After ethanol removal by washing with ice-cold PBS, the cells were resuspended in 1 mL of a propidium iodide (PI) staining solution (4% PI, 2 μg/mL RNase in PBS) and incubated for 30 min at 37 °C. The cells were subjected to cell cycle analysis using FACSCantoII (Becton, Dickinson and Company, San Jose, CA, USA) according to the manufacturer’s instructions. Analysis of cell cycle populations was performed using the BD Pro software.

### Animal experiments

C57BL/6 mice (male, 6-week-old) were purchased from Jung-Ang Lab Animal, Inc. (Seoul, South Korea). The animals were housed in a conventional state under adequate temperature (21–23 °C) and humidity (40–60%) control, with a 12-h light/dark cycle, and were given free access to food and water. C57BL/6 mice were fed a normal diet (ND) or an HFD for six weeks. Then, the HFD-fed mice were randomly divided into the following three groups (n = six per group): an HFD (distilled water-treated) group, HFD + low-dose GN (2 mg/kg of body weight) group, and HFD+high-dose GN (10 mg/kg of body weight) group. The experimental diets were the AIN93G-based on the High-fat diet containing 60% kcal fat and the control diet containing 10% kcal fat. GN was dissolved in distilled water and administered orally three times a week for eight weeks. The animal protocol used in this study was reviewed and approved by the Pusan National University’s Institutional Animal Care and Use Committee in accordance with the established ethical and scientific care procedures (approval number: PNU-2015–0940).

### Histological analysis

Epididymal adipose and liver tissues isolated from the mice were dissected and fixed in 10% buffered formalin. Fixed tissues were embedded in paraffin, and 5-μm sections were prepared using a frozen microtome (HM560H, Microm Laboratory, Walldorf, Germany). The sections of epididymal adipose were stained with hematoxylin and eosin (H&E), and the sections of liver tissue were stained with ORO, subjected to photomicroscopic observation.

### Analysis of serum biochemical parameters

After starvation for 12 h, the mice were sacrificed. Blood samples were collected and centrifuged at 1,000 × *g* for 15 min at 4 °C to obtain serum, and the serum was stored at −80 °C until analysis. Concentrations of glucose, triglycerides, and total cholesterol were determined using commercial analysis kits (Asan Diagnostics).

### Statistical analysis

All data were presented as the mean ± standard error of the mean (SEM). The statistical significances between various group was determined by one-way ANOVA analysis of variance, followed by Tukey’s test. Values were considered statistically significant at *P* < 0.05.

## Additional Information

**How to cite this article:** Jang, M.-K. *et al*. Gomisin N inhibits adipogenesis and prevents high-fat diet-induced obesity. *Sci. Rep.* **7**, 40345; doi: 10.1038/srep40345 (2017).

**Publisher's note:** Springer Nature remains neutral with regard to jurisdictional claims in published maps and institutional affiliations.

## Supplementary Material

Supplemental Table and Figures
