# Supplementary material for: Gomisin N inhibits adipogenesis and prevents high-fat diet-induced obesity
Source: Sci Rep. 2017 Jan 9;7:40345. doi: 10.1038/srep40345 (PMC5220372; doi:10.1038/srep40345)
Supplement: Supplemental Table and Figures [file srep40345-s1.pdf]

Supplementary information of the manuscript entitled “Gomisin N inhibits adipogenesis and prevents high-fat diet-induced obesity” (manuscript number, SREP-16-37462A) (authors : Min-Kyung Jang, Ye-RangYun, Ji-Hyun Kim, Mi-Hee Park, Myeong Ho Jung)

Supplementary Table S1. List of primers for q- PCR

Gene	Forward primer	Reverse primer
PPARγ	GTGCCAGTTTCGATCCGTAGA	GGCCAGCATCGTGTAGATGA
C/EBPα	CAAGAACAGCAACGAGTACCG	GTCACTGGTCAACTCCAGCAC
aP2	ACACCGAGATTTCCTTCAAACT	CCATCTAGGGTTATGATGCTCTTC
FAS	AGGTGGTGATAGCCGGTATGT	TGGGTAATCCATAGAGCCCAG
CyclinA	TGATGCTTGTCAAATGCTCAGC	AGGTCCTCCTGTACTGCTCAT
CyclinD	TGCCATCCATGCGGAAA	AGCGGGAAGAACTCCTCTTC
CDK2	CCTGCTTATCAATGCAGAGGG	TGCGGGTCACCATTTCAGC
CDK6	GCATCGTGATCTGAAACCGC	ATAGCTGGACTGGAGCAGGA
C/EBPβ	AAGCTGAGCGACGAGTACAAGA	GTCAGCTCCAGCACCTTGTG
JMJD2B	GGCCAAGATCATTCCACCCA	CCCACAGTCATGGCCTTCTT
Cdc25	ACCCCAAAATGTTGCCTTGA	AGTAAGCGGAGAGGCAGACATC
Cdc451	ACTGCTCACTCATGGAGGGC	GCAGACTCAAGGATGCTGGC
Mcm3	TGACCGTGGAGTGGTTTGC	GGCTGTGCGGTCCATGTC
MLL3	CAAGCCTTATTTGATTCCAC	GTTCTTCCATTTGGCATATT
MLL4	CATGGTGCCTGAAGATGT	TCTCTGATGCTGATGACGTA
G9a	GTTCCTTGTCTCCCCTCCCAG	GGAAGGGTCTCCTCACTCTGA

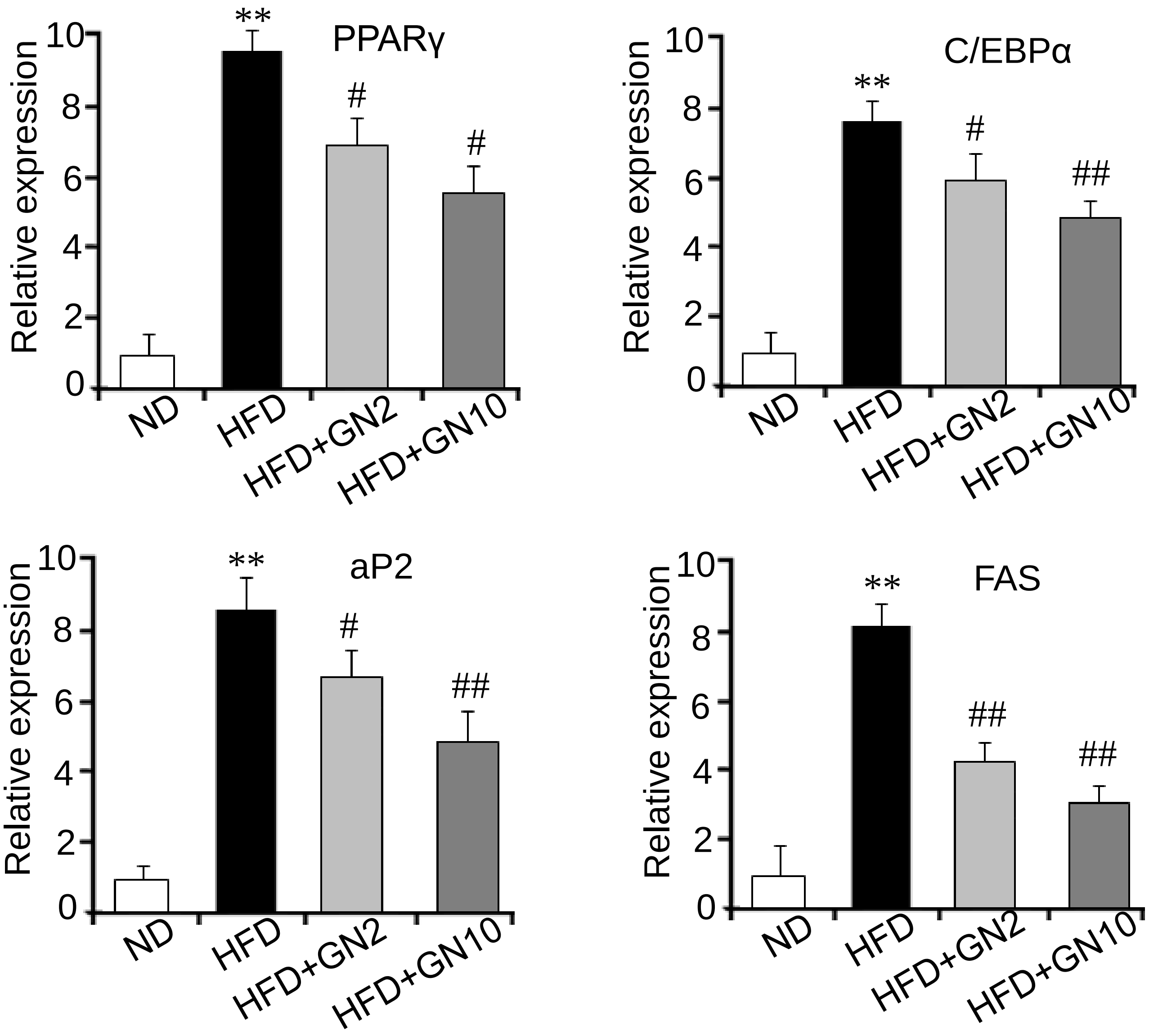

Supplemental Figure S1. Gomisin N (GN) downregulates adipogenic genes in HFD-induced obese mice. (A) Total RNA was isolated from adipose tissues of the mice, and the expression of adipogenic genes was determined by qPCR. The data are presented as the mean ± SEM for six mice. $^{**}P < 0.01$ vs. ND fed mice. $^{\#}P < 0.05$, $^{\#\#}P < 0.01$ vs. HFD fed mice alone.

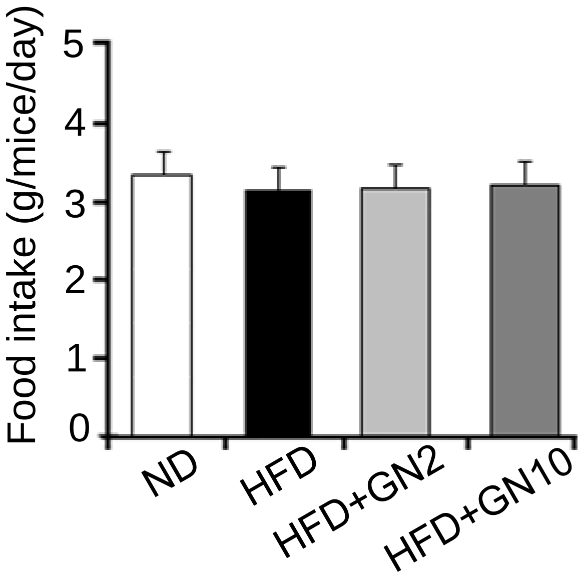

Supplemental Figure S2. Effects of Gomisin N on food intake. The food intake was measured twice a week for 8 weeks after oral administration of GN to HFD obese mice and then the average daily food intake was calculated.
